# Inequalities in diet quality by socio-demographic characteristics, smoking, and weight status in a large UK-based cohort using a new UK diet quality questionnaire-UKDQQ

**DOI:** 10.1017/jns.2024.60

**Published:** 2024-10-10

**Authors:** Kath Roberts, John Stephenson, Michelle Holdsworth, Clare Relton, Elizabeth A. Williams, Janet Elizabeth Cade

**Affiliations:** 1 Department of Health Sciences and the Hull York Medical School, University of York, York, UK; 2 Sheffield Centre for Health and Related Research, School of Medicine and Population Health, University of Sheffield, Sheffield, UK; 3 School of Human & Health Sciences, University of Huddersfield, Huddersfield, UK; 4 UMR MoISA (Montpellier Interdisciplinary centre on Sustainable Agri-food systems), (Univ Montpellier, CIRAD, CIHEAM-IAMM, INRAE, Institut Agro, IRD), Montpellier, France; 5 Wolfson Institute for Population Health, Queen Mary University, London, UK; 6 School of Food Science and Nutrition, University of Leeds, Leeds, UK

**Keywords:** Dietary assessment, Dietary ill-health, Dietary patterns, Diet quality, Diet quality questionnaire, Disparities, Inequalities, Smoking, Socio-economic, Weight

## Abstract

The aim of this study was to explore the associations between diet quality, socio-demographic measures, smoking, and weight status in a large, cross-sectional cohort of adults living in Yorkshire and Humber, UK. Data from 43, 023 participants aged over 16 years in the Yorkshire Health Survey, 2^nd^ wave (2013–2015) were collected on diet quality, socio-demographic measures, smoking, and weight status. Diet quality was assessed using a brief, validated tool. Associations between these variables were assessed using multiple regression methods. Split-sample cross-validation was utilised to establish model portability. Observed patterns in the sample showed that the greatest substantive differences in diet quality were between females and males (3.94 points; P < 0.001) and non-smokers vs smokers (4.24 points; P < 0.001), with higher diet quality scores observed in females and non-smokers. Deprivation, employment status, age, and weight status categories were also associated with diet quality. Greater diet quality scores were observed in those with lower levels of deprivation, those engaged in sedentary occupations, older people, and those in a healthy weight category. Cross-validation procedures revealed that the model exhibited good transferability properties. Inequalities in patterns of diet quality in the cohort were consistent with those indicated by the findings of other observational studies. The findings indicate population subgroups that are at higher risk of dietary-related ill health due to poor quality diet and provide evidence for the design of targeted national policy and interventions to prevent dietary-related ill health in these groups. The findings support further research exploring inequalities in diet quality in the population.

## Introduction

An unhealthy diet is one of the four leading behavioural causes of years of life lost in England alongside smoking, physical inactivity, and alcohol consumption.^(1)^ Poor quality diet is associated with obesity, type 2 diabetes, cardiovascular diseases, and some cancers and estimates of the economic cost of the risk of chronic disease on the NHS suggest that poor diet is the behavioural risk factor with the highest impact.^(2)^ Prevalence of these diseases is unequally distributed in the UK^(1,3,4)^ and socially patterned differences in dietary intake may therefore be a significant contributor to health inequalities.^(5)^ Compared with people living in the least deprived decile of local authority areas, people living in the most deprived areas are more likely to die from preventable cancers, preventable heart disease and are more likely to have type 2 diabetes. Children in Year 6 living in the most deprived communities are around twice as likely to be living with obesity compared with those living in the least deprived.^(6)^

These inequalities in diet-related ill health are reflected in variations in population-level dietary intake. Amongst UK children and adults, average consumption of total fat, saturated fat, and free sugars exceeds recommended levels while average consumption of fibre is too low.^(7)^ Whilst this is observed across age groups and sexes there is clear evidence that the extent to which recommendations are not met is socially distributed.^(8)^ Higher socio-economic groups tend to eat a greater number of daily grams of fruit and vegetables and fewer daily grams of red and processed meat and non-milk extrinsic sugars.^(5)^ On average adults in the UK with higher incomes consume a greater amount of fruits and vegetables, oily fish, fibre and a lesser amount of sugary drinks and free sugars.^(9)^ Children from families in the lowest 20% of income consume around a third less fruits and vegetables, three quarters less oily fish, and a fifth less fibre per day than children from the most well off 20%.^(10)^ Analysis of empirically derived dietary patterns characterised by higher consumption of fruit, vegetables, and oily fish were more likely to be consumed by higher socio-economic status groups whereas lower socio-economic status groups were more likely to consume dietary patterns characterised by snacks, fast foods and sugary drinks.^(11)^

Whilst UK studies show clear variation in consumption of individual foods and nutrients across socio-economic groups, exploration and analysis of dietary patterns and composite measures of diet quality can be a better indicator of habitual dietary intake and show stronger associations with health outcomes.^(12)^ This method examines diet as a multidimensional exposure, examining relationships with the whole diet and health rather than individual foods, food groups, or nutrients. However, there is a paucity of studies from the UK that explore dietary quality in the population.

Therefore, the aim of this study was to analyse the relationship between diet quality and its association with socio-economic and demographic characteristics, smoking status, and weight status in a large UK adult cohort. The regional population-based Yorkshire Health Study was used for this purpose. Diet quality was evaluated using a new, brief diet quality assessment tool developed for pragmatic application in large-scale surveys and developed and validated in a representative UK population.^(13)^

## Methods

### The Yorkshire health study

The aim of the Yorkshire Health Study was to collect information on the residents from the Yorkshire and Humber region in England to inform local health-related decision making.^(14)^ In the first phase, data were collected from 27,813 individuals aged 16–85 years (15.9% response rate) registered with GP surgeries between 2010 and 2012, in South Yorkshire. The second phase expanded to cover the Yorkshire & Humberside Government Office Region. In this second phase, data were collected from an additional 43,023 individuals between 2013 and 2015 via NHS Trusts, supported by the NIHR Clinical Research Network. A regional media campaign was also used to invite residents to sign up to join the cohort and to complete an online or paper health questionnaire. The questionnaire was used to capture demographic information on sex, age, socio-economic status, employment status, and deprivation level. Data were also collected relating to health related behaviours such as smoking status, height, and weight. BMI was calculated from self-reported valid height and weight data. Data on diet quality were collected in the 2^nd^ phase of YHS data collection using a 13 item Diet Quality Questionnaire (UK-DQQ). This dietary quality assessment tool was designed for pragmatic application and to be brief, low participant burden and easy to analyse and interpret.^(13)^

### Ethical standards

This study was conducted according to the guidelines laid down in the Declaration of Helsinki and all procedures involving research study participants were approved by the Leeds East National Health Service (NHS) Research Ethics Committee (ref: 09/H1306/97). An information sheet was sent to participants along with the questionnaire. Consent was implied if a questionnaire was returned to the researchers.

### Assessment of diet quality

Thirteen questions relating to the frequency of intake of specific foods were included in the Yorkshire Health Study questionnaire, the UK Diet Quality Questionnaire (UK-DQQ). Eleven food items (oily fish; wholemeal breads; salad and raw vegetables; bacon, ham, sausages, and burgers; sugary drinks; chips; biscuits; cakes and pastries; crisps and savoury snacks; white breads; coated or fried chicken; beer, lager, or cider) were included using a short ‘Food Frequency Questionnaire’ design. Fruit and vegetable (not raw) intake was assessed using an adapted version of an existing validated two question fruit and vegetable intake screener.^(15)^ The foods included in the questionnaire were generated from empirical dietary patterns analyses undertaken in data from the National Diet and Nutrition Survey 2008–2012.^(11)^ The questionnaire demonstrated that it was predictive of diet quality as measured by a composite measure with a validated Nutrient-based Diet Quality Score that was validated against blood and urine biomarkers of nutritional status and nutrient intake.^(15)^ The design of the UK-DQQ was intended to minimise participant burden, maximise accessibility and acceptability, be easy to analyse and interpret and thus practical for application in population level surveillance by public health professionals as well as academic researchers.

The scoring for the questions was developed to provide a simple method of analysing responses quantitatively, aiding interpretation and facilitating comparisons of patterns of diet quality across population subgroups. The scoring method reflects the associations that were observed in previous analyses between each food item and diet quality as defined by the Nutrient-based Diet Quality Score.^(15)^ Foods that are positively associated with diet quality such as fruit and vegetables are given greater scores for greater levels of consumption. Foods that are negatively associated with diet quality such as crisps and sugary drinks are given lower scores for greater levels of consumption. For full details of the scoring method please see the Supplementary Material.

### Statistical analyses

Diet quality scores were derived from participants who provided 50% or more valid responses to the 13 food-related items included in the derivation of the UK-DQQ. Data relating to participant gender, age, deprivation (measured by the Index of Multiple Deprivation (IMD) decile), weight category (categorised as *Underweight* (BMI <18 kg/m^2^); *Healthy weight* (18 kg/m^2^ ≤ BMI <25 kg/m^2^); *Overweight* (25 kg/m^2^ ≤ BMI <30 kg/m^2^), *Obese* (30 kg/m^2^ ≤ BMI <40 kg/m^2^), and *Severely obese* (BMI ≥40 kg/m^2^), smoking status (categorised as *current daily smoker*, *current occasional smoker*, *former daily smoker*, *former occasional smoker*, *never smoked*) and employment status (categorised as *unemployed*, *employed in sedentary occupation*, *employed in occasionally physical occupation*, *employed in physical occupation*, *employed in vigorous physical occupation*) was extracted from the Yorkshire Health Study database for analysis and relation to diet quality.

All variables were checked for errors, and cleaning procedures utilised if necessary. The cleaned sample was summarised descriptively. Variables with large amounts of missing data were tested for nature of data missingness. The distribution of all scores was inspected visually, and summary diet quality score statistics were derived for the entire sample and for each category in the categorical factors of interest. The relationship between diet quality scores and each of the demographic factors of interest in turn was investigated initially in a series of unadjusted screening analyses. Factors that appeared to be substantively related to the outcome in the uncontrolled models were carried forward for inclusion in a subsequent main effects multiple regression model. Formulation of the modelling of categorical factors in the multiple model was informed by inspection of plots of diet quality scores by category for linearity of relationship, with specific categories combined as necessary. P-values, parameter estimates, and associated confidence intervals (CIs) were determined for each included variable.

The multiple model was cross-validated using the split-sample method. A randomly selected 80% sample of the data was the training sample. Predicted values from this model were correlated against diet quality scores on this sample and the corresponding 20% validation sample. Similarities in the evaluated correlations were taken as indicative of good model portability.

## Results

### Descriptive and exploratory analysis

Dietary data were elicited from 43,023 participants with valid diet quality scores obtained from 41,235 respondents (95.8% of the total sample). The mean age of responders was 47 years (range 16–106), 69.0% were female, 54.8% had never smoked, 30.7% were not currently working (i.e. unemployed, retired) and 42.4% of the population self-reported weight and height that placed them in the ‘healthy weight’ category. Postcode was reported by 73.9% of the cohort, allowing IMD decile to be derived.

Diet quality scores were normally distributed with a mean score of 64.8 (SD 2.72). Theoretical minimum and maximum scores were 20 and 100; with higher scores indicating a better quality dietary pattern. The sample as a whole had generally low variability. A small number of outliers were observed; the range of values obtained from the sample was from 22 to 98; near to the tool minima and maxima. Levels of missing data were generally low on all factors except IMD, due to over a quarter of respondents failing to provide valid postcodes, from which IMD deciles could be calculated. Separate variance *t*-tests conducted on the IMD data revealed no evidence that missing data was not missing at random. Hence complete case analysis was conducted on the data without recourse to data imputation. The descriptive characteristics of the sample are summarised in Table 1.

**Table 1.** Descriptive summary of sample characteristics. Data are shown as %. Age is shown as mean (sd) and range

Variable	Frequency (valid %)
Gender (*n* = 42,477)	
Male	13,148 (31.0)
Female	29,329 (69.0)
IMD decile (*n* = 23,904)	
1 (most deprived)	3128 (13.1%)
2	1849 (7.7%)
3	2150 (9.0%)
4	2080 (8.7%)
5	2241 (9.4%)
6	2417 (10.1%)
7	2703 (11.3%)
8	2502 (10.5%)
9	2366 (9.9%)
10 (most affluent)	2468 (10.3%)
Employment category (*n* = 40,966)	
Unemployed	12,572 (30.7%)
Employed: sedentary occupation	12,509 (30.5%)
Employed: limited physical occupation	7967 (19.4%)
Employed: physical occupation	7279 (17.8%)
Employed: vigorous physical occupation	639 (1.6%)
Age (years) (*n* = 41,630)	47.1 (17.7; 16–106) Mean (SD; range)
Smoking status (*n* = 41,690)	
Daily smoker	4069 (9.8%)
Occasional smoker	1822 (4.4%)
Former daily smoker	7790 (18.7%)
Former occasional smoker	5146 (12.3%)
Never smoked	22,863 (54.8%)
Weight category (*n* = 38,229)	
Underweight (BMI <25 kg/m^2^)	527 (1.38%)
Healthy weight (BMI 18–25 kg/m^2^)	16,214 (42.4%)
Overweight (BMI 25–30 kg/m^2^)	12,313 (32.2%)
Obese (BMI 30–40 kg/m^2^)	8009 (21.0%)
Severely obese (BMI >40 kg/m^2^)	1166 (3.1%)

Diet quality scores in subgroups defined by the categorical variables are summarised in Table 2. Significance levels from one-way analysis of variance (ANOVA) tests on each variable, conducted as screening tests to assess the variables for inclusion in the subsequent multiple model, are also provided.

**Table 2.** Diet Quality Questionnaire scores by gender, deprivation level, smoking status, employment category and weight category. Data are shown as mean score (sd) and P value

Variable	Diet quality score (SD)	P-value^a^
Gender (*n* = 40,778)		<0.001
Male (*n* = 12,495)	62.5 (9.19)	
Female (*n* = 28,283)	65.9 (9.10)	
IMD decile (*n* = 23,098)		<0.001
1 (*n* = 2938)	62.9 (9.80)	
2 (*n* = 1780)	63.8 (9.59)	
3 (*n* = 2085)	64.4 (9.39)	
4 (*n* = 1994)	64.8 (8.98)	
5 (*n* = 2151)	65.2 (8.91)	
6 (*n* = 2359)	65.6 (9.11)	
7 (*n* = 2635)	66.0 (9.00)	
8 (*n* = 2438)	66.0 (8.84)	
9 (*n* = 2305)	66.7 (8.85)	
10 (*n* = 2413)	67.0 (8.39)	
Smoking status (*n* = 40,593)		<0.001
Daily smoker (*n* = 3934)	59.3 (9.75)	
Occasional smoker (*n* = 1783)	62.4 (9.24)	
Former daily smoker (*n* = 7560)	65.2 (8.97)	
Former occasional smoker (*n* = 5022)	65.9 (8.86)	
Never smoked (*n* = 22,294)	65.7 (9.00)	
Employment category (*n* = 39,996)		<0.001
Unemployed (*n* = 11,956)	65.4 (9.37)	
Employed: sedentary occupation (*n* = 12,415)	65.4 (8.74)	
Employed: limited physical occupation (*n* = 7831)	64.8 (9.24)	
Employed: physical occupation (*n* = 7175)	63.8 (9.63)	
Employed: vigorous physical occupation (*n* = 619)	60.7 (10.1)	
Weight category (*n* = 36,888)		<0.001
Underweight (BMI <18 kg/m^2^) (*n* = 498)	63.6 (10.6)	
Healthy weight (BMI 18 to 25 kg/m^2^) (*n* = 15,961)	65.5 (9.32)	
Overweight (BMI 25 to 30 kg/m^2^) (*n* = 11,865)	65.1 (8.92)	
Obese (BMI 30 to 40 kg/m^2^) (*n* = 7710)	64.5 (9.13)	
Severely obese (BMI >40 kg/m^2^) (*n* = 1124)	64.5 (9.74)	

a P-values based on uncontrolled comparisons of diet quality scores across groups defined by levels of controlling variables.

Hence higher scores were recorded in females; in those from higher IMD deciles; in former smokers and non-smokers; in those with no or limited physical component to their occupation; and in those who were categorised as ‘healthy weight’ or ‘overweight’.

**Fig. 1.**
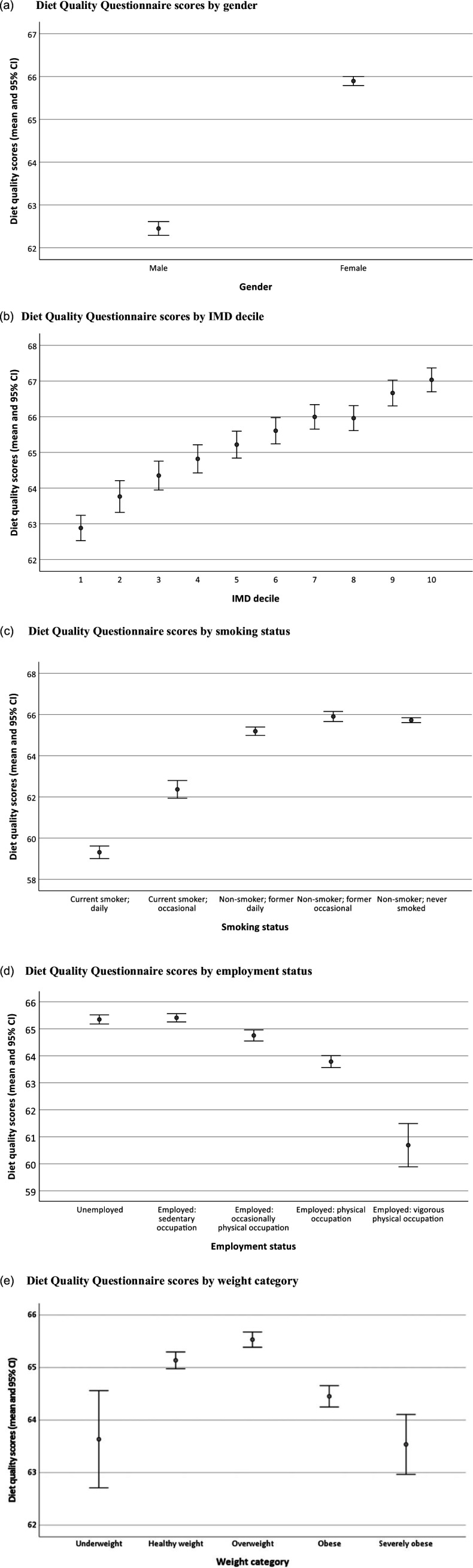
a–e: Subgroup diet quality scores by gender, IMD decile, smoking status, employment status, and weight category. Data are shown as means and 95% confidence intervals. (a) Diet Quality Questionnaire scores by gender. (b) Diet Quality Questionnaire scores by IMD decile. (c) Diet Quality Questionnaire scores by smoking status. (d) Diet Quality Questionnaire scores by employment status. (e) Diet Quality Questionnaire scores by weight category.

Subgroup diet quality scores are illustrated in Fig. 1.

While all associations tested in univariable screening analyses were significant at the 5% significance level, the substantive magnitude of effects varied. The gender effect was large, with females scoring 3.4 points more than males on the diet quality score scale. A near-monotonic increase in diet quality scores from the 1^st^ decile (most deprived) to the 10^th^ decile (least deprived) was observed, with substantive differences from 62.9 (decile 1) to 67.0 (decile 10). Little substantive difference was revealed in diet quality scores amongst the groups representing current non-smokers (including former occasional or daily smokers and those who have never smoked). Current daily smokers had the lowest dietary scores, while current occasional smokers were at an intermediate level between current daily smokers and non-smokers. The difference in diet quality scores between those of the lowest category (current daily smokers; 59.3) and the highest category (former occasional smokers; 65.9) was substantive. Diet quality scores of current daily smokers were about 2 standard deviations below the overall mean. A near-monotonic decrease in diet quality scores with increasing components of physical activity in employment was observed; however, not all differences between categories were substantive or significant. A monotonic decrease in diet quality scores with increasing weight category was observed for individuals categorised as ‘healthy weight’, ‘overweight’, ‘obese’, and ‘severely obese’. Underweight individuals had the lowest diet quality of all weight category groups.

### Multiple regression analysis

The screening analyses suggested that gender, age, socio-economic status (as measured by IMD decile), occupation group, smoking status, and weight category may be substantively related to diet quality scores. Inspection of plots derived from the smoking status, occupation status, and weight category suggested that the relationship between diet quality scores and the ordinal levels of these variables could not be assumed to be linear. *Ex-smoker* and *Non-smoker* smoking categories and the *Employed in sedentary occupation* and *Unemployed* occupation categories were hence combined for inclusion in the multiple model (becoming the reference category); in which the effect of levels of categorical variables was modelled using indicator variables. Weight category was modelled using a series of indicator variables compared to the reference category *‘healthy weight’.* A main effects multiple regression analysis conducted on the data revealed that all included factors and covariates were statistically significant (at the 5% significance level) except for employment in occasional physical occupation (compared with the reference group of unemployed or sedentary occupation) (Table 3).

**Table 3.** Multiple regression analysis of Diet Quality Scores with gender, age, socio-economic status (as measured by IMD decile), occupation group, smoking status and weight category. Data are shows as parameter estimate, 95% CI and P value

Variable	Parameter estimate	95% CI for estimate	P-value
Gender = female^a^	3.94	(3.69, 4.20)	<0.001
Age (years)	0.088	(0.080, 0.095)	<0.001
IMD decile	0.243	(0.202, 0.284)	<0.001
Employment status = occasional physical occupation^c^	–0.012	(–0.337, 0.313)	0.943
Employment status = physical occupation^c^	–0.565	(–0.899, –0.232)	0.001
Employment status = vigorous physical occupation^c^	–1.28	(–2.29, –0.278)	0.012
Current smoker^b^	–4.24	(–4.60, –3.88)	<0.001
Weight category = underweight^d^	–1.63	(–2.69, –0.583)	0.002
Weight category = overweight^d^	–0.706	(–0.986, –0.426)	<0.001
Weight category = obese^d^	–1.44	(–1.76, –1.12)	<0.001
Weight category = severely obese^d^	–2.59	(–3.27, –1.90)	<0.001

a Reference = male.

b Reference = former smoker or non-smoker.

c Reference = unemployed or sedentary occupation.

d Reference = healthy weight.

Controlling for other factors and covariates, the model revealed that females scored 3.94 more points on the UK-DQQ than males; each increasing IMD decile (i.e. decreasingly deprived) increased scores by 0.243 points; smokers score 4.24 points less than non-smokers; those whose employment involved physical activity scored 0.565 points less than those who were unemployed or in sedentary employment; those whose employment involved vigorous physical activity scored 1.28 points less than those who are unemployed or in sedentary employment; those categorised as ‘underweight’ score 1.63 points less than those categorised as ‘healthy weight’; those categorised as ‘overweight’ scored 0.706 points less than those of ‘healthy weight’; those categorised as ‘obese’ scored 1.44 points less than those of ‘healthy weight’; those categorised as ‘severely obese’ scored 2.59 points less than those of ‘healthy weight’; each year of age increased scores by 0.088 points. Differences in scores between subgroups may be interpreted in terms of the theoretical 80-point range of the tool (minimum of 20 to maximum of 100); hence, the largest individual effect noted (that of smoking) represents about a 5 percentage point difference between smokers and non-smokers.

Cross-validation procedures revealed that the model had good portability, with negligible reduction in correlation between predicted values and dietary scores when a model derived from the training sample (*r* = 0.159; P < 0.001) was applied to the validation sample (*r* = 0.151; P < 0.001).

## Discussion

The purpose of this study was to explore the relationship between patterns of diet quality, socio-demographic measures, smoking, and weight status in a large cross-sectional cohort of adults living in Yorkshire & Humber, UK. The main findings were that higher diet quality was associated with being female and the poorest diet quality was associated with being a current smoker. Diet quality scores were higher with increasing socio-economic status and age. Employment in a physically active job, being male, being of obese weight or underweight were each associated with lower diet quality. This provides evidence for diet quality being unequally distributed in the population and indicates population characteristics by which these inequalities are patterned. This provides a focus for targeted resource and intervention to prevent diet-related ill health in these sub groups.

The current study focuses on the relationship between diet quality and population characteristics. Whilst a direct comparison of our results with previous surveys is not possible due to methodological differences, the findings are consistent with observations in the UK where diet quality has been inversely associated with measures of socio-economic status and positively associated with age.^(11,16,17)^

Our results confirm findings from previous cross-sectional studies of an inverse association between diet quality and smoking status.^(18)^ It is well established that smokers have poorer micronutrient status, an association that is independent of dietary intake and likely explained by increased micronutrient requirements from increased oxidative stress of smoking.^(19)^ The observation that smokers have a poorer diet quality is likely to further exacerbate the risk of chronic disease associated with smoking.

In agreement with our results, studies in the UK have reported better diet quality in women than men.^(11,20)^ We identified a clear linear relationship between socio-economic status (as measured by the Index of Multiple Deprivation) and diet quality (as measured by the UK Diet Quality Questionnaire). Studies in the UK and globally have reported similar findings with better diet quality in more affluent households.^(21,22)^ A lower diet quality, including dietary patterns that were lower in fruit and vegetable consumption and lower in dietary diversity, has been associated with lower socio-economic status, lower income and food insecurity in a number of developed countries including the UK and the US.^(23–26)^ The cost of a healthy diet may account in part for the association between diet quality and socio-economic status,^(27–29)^ and the relationship between cost and diet quality is likely to be bidirectional.

Poorer diet quality was associated with obesity in this study. Finding which reflect those of other UK studies.^(22)^ Diet is known to be a major determinant of morbidity and mortality. Poor diet quality is associated with negative health outcomes across the lifecourse and greater risk of a number of chronic diseases including obesity, a range of cancers, type-2 diabetes, cardiovascular disease, frailty, and mortality risk,^(30–35)^ and more recently, risk and severity of COVID-19 infection.^(36)^

This study has several strengths. It was undertaken in a large, representative sample.^(37)^ Key findings in this study such as the associations between deprivation and diet quality and smoking and diet quality are consistent with expected observations and provide further evidence on health inequalities in the UK, an important and relevant topic for current public health policy makers. While a small amount of missing data was recorded on most variables in this study, there was no evidence that missing data were not missing at random. The model showed good cross-validation properties and would be expected to be applicable to further datasets with negligible reduction in correlation between predicted values and dietary scores.

The food items included in the UK-DQQ were developed from empirical and theoretical dietary pattern analyses conducted in the National Diet and Nutrition Survey, a nationally representative dataset of nutrient-level dietary data from UK adults.^(7,11)^ Dietary patterns take account of the synergistic relationship between nutrients and foods, and there is evidence that dietary patterns have stronger correlations with health outcomes than analysis of single nutrients or foods.^(12,38)^ The food items included in the UK-DQQ were associated with patterns of diet quality and were predictive of a validated Nutrient-based Diet Quality Score (see Appendix A).^(13,15,39)^ This is the first survey in which this dietary assessment tool has been applied and the findings suggest that it is a suitable tool for UK population-based studies and that it can detect differences in diet quality. The advantage of this dietary assessment tool over others is its brevity, with just 13 questions used to capture diet quality. In addition, its scoring methodology is simple to use and analyse and aids interpretation of findings by non-nutrition experts. This makes it practical for incorporation into population level surveys. The number of questions keeps the time for completion and respondent burden to a minimum and reduces the likelihood of survey fatigue. The tool provides an overall picture of the quality of the diet, rather than detailed nutrient intake. As the tool is independent of an individual’s energy intake, energy adjustments are not required, which reduces the chances of measurement error.^(40)^

The study has some limitations. A substantive proportion of variance in diet quality scores was unexplained by the analysed factors (adjusted R^2^ = 0.111 for the multiple model); suggesting that diet may also be related to additional factors not included in the current analysis. This is consistent with the fact that diet is a complex, multifaceted behaviour. Our analysis examined factors that determine diet quality separately and assumed the impact on diet quality was additive as no significant interactions were found. However, there is good evidence that certain health behaviours such as smoking and dietary intake cluster^(41)^ and whilst some characteristics may be additive it is possible that a combination of characteristics such as socio-economic status, sex, and obesity act synergistically.^(42)^ This could have significant implications for identification of at risk populations, policy development and intervention design.

The cohort in this study represents the population living in the Yorkshire and Humber region of the UK. The cohort was not a random sample and was largely self-selected. Nonetheless, taking into account the proportion of females in the survey, the prevalence of overweight and obesity is in line with what has been reported elsewhere, with the Health Survey for England reporting 63% of adults in the Yorkshire and Humber region are overweight or obese, compared with 56.3% in this survey.^(6)^ It is well recognised that people who participate in such surveys are likely to be better educated and be motivated to participate. Despite this probable self-selection there was good representation across all of the IMD deciles. We anticipate that self-selection would dampen associations; nonetheless, the significant variability in UK-DQQ across the data was apparent.

Further research in this cohort could consider the association between diet quality with use of health services and incidence of disease and long-term health conditions. The YHS is a longitudinal survey and participants have given permission for medical records to be examined. This could facilitate the prospective exploration between the relationship between diet quality and disease. Other factors known to influence diet quality include income, level of education, and household composition, and these factors could be considered in future analysis. It would also be useful to apply the UK-DQQ across different UK regions and in different ethnic groups.

## Conclusions

Analysis of diet quality in a large epidemiological dataset has revealed inequalities in patterns of diet quality. Females, non-smokers, those living in areas of lower deprivation yield the highest scores, indicative of a dietary pattern that is better quality. These findings highlight that men, smokers, younger people, and those in lower socio-economic groups are most at risk from diet related ill health. These findings reinforce the evidence for there being multiple layers of inequality in diet quality at population level that contribute to population level health inequalities. These findings are important for informing public health nutrition policy at national and local level and providing evidence for where public health resource and intervention should be directed in order to reduce widening health inequalities. The findings indicate that brief tools can usefully be used in large-scale self-reported surveys in the UK population to assess broad patterns of inequalities in diet quality.

The study findings support further research to understand the association between diet quality in the UK and a wider range of socio-demographic and socio-economic variables such as ethnic group, household composition, and food insecurity status; health-related variables such as physical activity levels and alcohol intake; place-based variables such as access to green space, food access, and access to public transport and other health outcomes such as long-term health conditions and disability, mental health, self-reported health, and self-reported quality of life. It also provides support for further research into the causal determinants of these inequalities in diet quality in different population sub-groups in order to inform intervention development and policy.

## Supporting information

Roberts et al. supplementary material Roberts et al. supplementary material

## Abbreviations

**BMI:** Body Mass Index; **IMD:** Index of Multiple Deprivation; **UK-DQQ:** UK Diet Quality Questionnaire; **YHS:** Yorkshire Health Study.

## Supplementary material

The supplementary material for this article can be found at https://doi.org/10.1017/jns.2024.60
