# Supplementary material for: Inequalities in diet quality by socio-demographic characteristics, smoking, and weight status in a large UK-based cohort using a new UK diet quality questionnaire-UKDQQ
Source: J Nutr Sci. 2024 Oct 10;13:e59. doi: 10.1017/jns.2024.60 (PMC11503710; doi:10.1017/jns.2024.60)
Supplement: Roberts et al. supplementary material [file S2048679024000600sup001.docx]

**Supplementary Material (to be made available online).**

**Figure 1. Foods characterising empirically derived dietary patterns in UK adults (foods in bold most strongly associated with dietary pattern and included in the UK-DQQ)**

| **Dietary Pattern** | **Foods with moderate/strong positive relationship to dietary pattern*** | **Foods with moderate/strong negative relationship to dietary pattern*** |
| --- | --- | --- |
| Fruit, vegetables and oily fish (FVOF) | **Fruit**  **Salad and other raw vegetables**  Yoghurt, fromage frais and dairy desserts  **Oily fish**  Nuts and seeds  Wine  **Vegetables (not raw)**  Tea, coffee and water  **Wholemeal bread** | **Chips (fried), roast potatoes and potato products**  **White bread**  Sugar, preserves and sweet spreads  Whole milk  White fish coated or fried |
| Snacks, fast food and fizzy drinks | **Crisps and savoury snacks**  **Coated chicken and turkey**  **Soft drinks (not low calorie)**  Soft drinks (low calorie)  Burgers and kebabs  Chocolate confectionery  Chips (fried), roast potatoes and potato products  Sugar confectionery  White bread | Tea, coffee and water  High fibre breakfast cereals  Other potatoes, potato salads and dishes  Fruit  **Vegetables (not raw)** |
| Sugary food and dairy (SFD) | Sugars, preserves and sweet spreads  **Buns, cakes, pastries and fruit pies**  **Biscuits**  Chocolate confectionery  Semi skimmed milk  Reduced fat spread  Tea, coffee and water  Cheese  Puddings  **White bread** | Wine |
| Meat, potatoes and beer | Savoury sauces, pickles, gravies and condiments  Other potatoes, potato salads and dishes  **Beer, lager, cider and perry**  White bread  Pork and dishes  **Bacon and ham**  Whole milk  Spirits and liqueurs  Butter |  |

**Scoring for Diet Quality Questionnaire**

**Figure 2. Foods positively associated with diet quality**

| Food | Never or occasionally | 1-3 times a week | 4-6 times a week | Daily | More than once a day |
| --- | --- | --- | --- | --- | --- |
| Fruit* | 2 | 4 | 6 | 8 | 10 |
| Vegetables* | 2 | 4 | 6 | 8 | 10 |
| Oily fish | 2 | 4 | 6 | 8 | 10 |
| Wholemeal breads, pittas, nans etc | 2 | 4 | 6 | 8 | 10 |
| Salad and raw vegetables | 2 | 4 | 6 | 8 | 10 |

Figure 3. Food negatively associated with diet quality

| Food | Never or occasionally | 1-3 times a week | 4-6 times a week | Daily | More than once a day |
| --- | --- | --- | --- | --- | --- |
| Bacon, ham, sausages and burgers | 5 | 4 | 3 | 2 | 1 |
| Sugary drinks | 10 | 8 | 6 | 4 | 2 |
| Chips | 5 | 4 | 3 | 2 | 1 |
| Biscuits, cakes and pastries | 5 | 4 | 3 | 2 | 1 |
| Crisps and savoury snacks | 5 | 4 | 3 | 2 | 1 |
| White breads, pittas, naans etc | 5 | 4 | 3 | 2 | 1 |
| Coated or fried chicken | 5 | 4 | 3 | 2 | 1 |
| Beer, lager or cider | 10 | 8 | 6 | 4 | 2 |
